# Feelings of Helplessness in ET: A Preliminary Analysis of Prevalence and Clinical Correlates

**DOI:** 10.5334/tohm.1295

**Published:** 2026-10-22

**Authors:** Elan D. Louis

**Affiliations:** 1 Department of Neurology, University of Texas Southwestern Medical Center, Dallas, Texas, USA; 2 Peter O’Donnell Jr. Brain Institute, University of Texas Southwestern Medical Center, Dallas, TX, USA; 3 Department of Epidemiology, Peter O’Donnell Jr. School of Public Health, University of Texas Southwestern Medical Center, Dallas, TX, USA

**Keywords:** essential tremor, clinical, helplessness

Essential tremor (ET) is a highly prevalent disease [1] or family of diseases [2] whose primary motor feature is kinetic tremor [3]. In many patients, the tremor may become both severe [4] and disabling [5], and in patients with long-standing disease, the tremor may be incapacitating [6]. Studies have reported that 15%–25% of patients with ET are forced to retire prematurely, and 60% choose not to apply for a job or promotion because of uncontrollable shaking [7,8]. Tremor medications have been only partially successful at ameliorating patient’s symptoms, leaving a high burden of under-treated and untreated disease [9].

The impact of tremor among patients with ET is multi-dimensional, with patients experiencing both increased frailty [10] and lower morale [11] relative to age-matched controls; furthermore, increased caregiving intensity [12] and caregiver burden [13] have been reported in some cases. Given this clinical and therapeutic scenario, it is understandable that ET patients may at times feel helpless (i.e., experience feelings of having reduced control over aversive outcomes). To our knowledge, little is understood about helplessness in patients with ET. Hence, the goal of these preliminary analyses was to assess the prevalence of reported helplessness as well as its clinical correlates in indivduals with ET.

ET cases were enrolled in a prospective, longitudinal, nationwide study of older adults with ET (Clinical Pathological Study of Cognitive Impairment in Essential Tremor [COGNET]; National Institutes of Health Awards #R01 NS086736 and #R01 NS145265), described in detail [14]. To date, cases reside in 43 US states, having responded to an advertisement to enroll in this cohort [14]. The Yale University, Columbia University, and University of Texas Southwestern Medical Center Institutional Review Boards approved the study protocol. All cases provided written, informed consent. During the in-home baseline evaluation, ET cases were asked, “Do you often feel helpless?”, which was one of 30 questions in the Geriatric Depression Scale (GDS) [15].

Data were available on 101 ET cases whose mean age was 79.8 ± 5.3 years (range = 65–96 years) and among whom 60 (59.4%) were women. Their mean total tremor score was 21.1 ± 6.1 (highest potential value = 36), indicating moderate action tremor on average, and 67 (66.4%) were taking mediation to treat their tremor. Twelve (11.9%, 95% confidence interval = 6.3% to 19.8%) of 101 reported that they often felt helpless.

ET cases who reported feelings of helplessness had a higher mean total tremor score than did those who did not (25.0 ± 6.4 vs. 20.7 ± 5.9, Student’s t test, p = 0.04). The two groups did not differ with respect to tremor duration (median durations = 23.0 vs. 31.0 years, Mann Whitney test, p = 0.2). The total burden of medical illnesses as assessed using the Cumulative Illness Rating Scale Score (range = 0–42 [highest level of comorbidity] [16] differed to a marginal degree (medians scores = 8.7 vs. 6.6, Mann Whitney test, p = 0.10), as did the proportion of those who had been prescribed but had stopped tremor medication (7 [58.3%] vs. 27 [30.3%], Fisher’s Exact test = 0.099). We removed the one helplessness question from the GDS and then assessed the correlation between this modified (i.e., 29-item rather than 30-item) GDS and the helplessness question. The Spearman’s rho = 0.35, p = 0.002.

A 2020 study of 60 ET cases from the same parent study focused on the issue of demoralization, a manifestation of psychological distress that is similar to but distinct from depression [17]. That study used the Kissane Demoralization Scale (KDS-II) [18], which included 16 questions that queried patients about feelings of demoralization. One of the sixteen questions was, “I feel that I cannot help myself”, which is similar although not identical to the question we used in the current study. First, the wording was different. Second, the question was part of an instrument that focused on *psychological* issues. Indeed, the other 15 questions in the KDS-II focus on psychic and emotional issues – “my role in life has been lost”, “I have a lot of regret about my life”, I would rather not be alive”, and “I feel quite isolated and alone”. Therefore, while helplessness was queried in the 2020 study, it was interpreted more as a psychological than a physical construct in that study.

In summary, one in ten ET patients report feelings of helplessness, and this feeling tracks with tremor severity. These are preliminary analyses, and it is important to follow these with more in-depth analyses of helplessness in ET, using more detailed and nuanced questions. More specifically, future work should employ additional questions about helplessness and delve into the specific activities of daily living that are most likely to be associated with feelings of helplessness. Leaving future work aside, the signal we observe suggests that there is an additional dimension to ET, and one that clinicians should be consider when caring for their patients with ET, especially as helplessness can contribute to distress, anxiety, withdrawal, physical disease and death [19].
